# Photosynthetic performance and nutrient uptake under salt stress: Differential responses of wheat plants to contrasting phosphorus forms and rates

**DOI:** 10.3389/fpls.2022.1038672

**Published:** 2022-11-10

**Authors:** Aicha Loudari, Asmae Mayane, Youssef Zeroual, Gilles Colinet, Abdallah Oukarroum

**Affiliations:** ^1^ Plant Stress Physiology Laboratory–AgroBioSciences, Mohammed VI Polytechnic University (UM6P), Benguerir, Morocco; ^2^ Terra Research Center, Gembloux Agro Bio Tech Faculty, Liege University (ULIEGE), Gembloux, Belgium; ^3^ High Throughput Multidisciplinary Research Laboratory, Mohammed VI Polytechnic University (UM6P), Benguerir, Morocco

**Keywords:** durum wheat, polyphosphate, phosphorus, photosynthetic performance, salinity, nutrient uptake

## Abstract

Salt stress impacts phosphorus (P) bioavailability, mobility, and its uptake by plants. Since P is involved in many key processes in plants, salinity and P deficiency could significantly cause serious damage to photosynthesis, the most essential physiological process for the growth and development of all green plants. Different approaches have been proposed and adopted to minimize the harmful effects of their combined effect. Optimising phosphorus nutrition seems to bring positive results to improve photosynthetic efficiency and nutrient uptake. The present work posed the question if soluble fertilizers allow wheat plants to counter the adverse effect of salt stress. A pot experiment was performed using a Moroccan cultivar of durum wheat: Karim. This study focused on different growth and physiological responses of wheat plants grown under the combined effect of salinity and P-availability. Two Orthophosphates (Ortho-A & Ortho-B) and one polyphosphate (Poly-B) were applied at different P levels (0, 30 and 45 ppm). Plant growth was analysed on some physiological parameters (stomatal conductance (SC), chlorophyll content index (CCI), chlorophyll a fluorescence, shoot and root biomass, and mineral uptake). Fertilized wheat plants showed a significant increase in photosynthetic performance and nutrient uptake. Compared to salt-stressed and unfertilized plants (C+), CCI increased by 93%, 81% and 71% at 30 ppm of P in plants fertilized by Poly-B, Ortho-B and Ortho-A, respectively. The highest significant SC was obtained at 45 ppm using Ortho-B fertilizer with an increase of 232% followed by 217% and 157% for both Poly-B and Ortho-A, respectively. The Photosynthetic performance index (PI_tot_) was also increased by 128.5%, 90.2% and 38.8% for Ortho-B, Ortho-A and Poly B, respectively. In addition, Poly-B showed a significant enhancement in roots and shoots biomass (49.4% and 156.8%, respectively) compared to C+. Fertilized and salt-stressed plants absorbed more phosphorus. The P content significantly increased mainly at 45 ppm of P. Positive correlations were found between phosphorus uptake, biomass, and photosynthetic yield. The increased photochemical activity could be due to a significant enhancement in light energy absorbed by the enhanced Chl antenna. The positive effect of adequate P fertilization under salt stress was therefore evident in durum wheat plants.

## 1 Introduction

It is well known that soil salinity causes an imbalance in mineral uptake and plant nutrition (Shabala and Munns, 2017; Behdad et al., 2021) and the initial action of salinity is revealed by a decrease in water absorption capacity in the rooting zone (Zhao et al., 2020). This nutritional disorder induces changes in the plant at morphological, physiological, and metabolic levels (Kumari et al., 2022). The response of plants to an excess of sodium ions (Na^+^), the most important salinity-causing substance, is complex and involves a cascade of mechanisms to reduce the adverse effects of Na^+^ (Chavarria et al., 2020). Furthermore, tolerance to high salinity may be expected to vary with plant species and different growth stages of plants (Xue et al., 2009). There is evidence that high salt stress provokes a multitude of negative plant responses such as induction of osmotic stress generating reactive oxygen species (ROS) (Kumar et al., 2017; Hasanuzzaman et al., 2021), reduction in photosynthesis (Chaves et al., 2011; Oukarroum et al., 2015; Rahimi et al., 2021), degradation of photosynthetic pigments (Muhammad et al., 2021), and reduction in stomatal conductance (Lotfi et al., 2020). However, salinity adaptive in plants is activated by a series of defence mechanisms at all plant levels as well as at anatomical levels (Chavarria et al., 2020). In the current context of intensive agriculture and the increasing effects of salinity which appear in several regions in the world (Pulido-Bosch et al., 2018), Different approaches have been proposed and adopted to minimize the adverse effects of salt stress on plant development and productivity. Among the strategies reported is the use of arbuscular mycorrhizal fungi (AMF) (Elhindi et al., 2017; Li et al., 2020), inoculation with plant-growth-promoting rhizobacteria (PGPR)  (Ilangumaran and Smith, 2017; Tirry et al., 2021), nutritional supplementation of silicon (Altuntas et al., 2018; Muhammad et al., 2022), the addition of organic soil amendment (Yang et al., 2020; Kumari et al., 2022; Xiao et al., 2022) and exogenous application of hormones (Kaya et al., 2013). Regarding phosphorus (P) being the most important nutrient after nitrogen (N) for plant growth and development, salt stress has been reported to impact its bioavailability and mobility in the plant-soil continuum, and therefore root uptake (Demiral, 2017; Khan et al., 2018; Bouras et al., 2021; Bouras et al., 2022). The P deficiency impacts all vital processes: respiration, photosynthesis and plant growth and development (Carstensen et al., 2018). Leaves become smaller, thinner, and change colour into blue-green due to carbohydrate accumulation and the delay in protein synthesis (Meng et al., 2021). Similarly, the O_2_ absorption speed is decreased, and the enzyme activity involved in respiration is altered (Meng et al., 2021). P deficiency and salinity significantly alter the photosynthesis machinery and the mesophyll metabolism in different ways (Chaves et al., 2011; Kalaji et al., 2018) but their effects on photosynthetic metabolic processes and the ultrastructure of organelles are additional and important (Meng et al., 2021). The salinity effect is directly attributed to the limitation in gas diffusion due to the stomatal closure (Zribi et al., 2011; Asrar et al., 2017) and to the high accumulation of Na+ and Cl- in the chloroplasts which damages the membrane of thylakoids (Ashraf and Harris, 2013), while P deficiency disturbs ultimately the CO2 assimilation since P is implicated in the transport of fixed carbon from chloroplasts to the cytosol with its triose-phosphate form (Rychter et al., 2018). Hence, any alteration of the photosynthesis mechanism caused either by P deficiency or by salinity may reduce the overall photosynthetic capacity of the plant (Kalaji et al., 2016; 2018). This reduction causes a decline in crop yield which affects food security around the world (Muhammad et al., 2021).

A better understanding of the photosynthetic processes could therefore help to assess the potential of key photosynthetic components under the combined effect of salinity and P deficiency to balance the photosynthetic light reactions with downstream metabolism and a higher crop yield. Optimising phosphorus nutrition seems to bring positive results (Khan et al., 2018; Mohamed et al., 2021; Bouras et al., 2021; 2022). However, the P use efficiency in salt-stressed plants differs depending on the severity of stress in the rhizosphere (Zhao et al., 2020). Several investigations have been conducted to understand the effects of salt stress and phosphorus interaction in different plant species, degree of salinity severity, and growing conditions (Abbas et al., 2018). Most results agreed that salinity reduces P accumulation in plant tissues (Khan et al., 2018; Belouchrani et al., 2020).

However, phosphorus uptake by plants, in the form of phosphate ions, depends on soil physicochemical parameters (Pereira et al., 2020), the application method and its frequency (Rady et al., 2018; Chtouki et al., 2022), the root exudation and architecture (Khourchi et al., 2022a), and the rhizosphere microbial activity (Wahid et al., 2020). In this regard, different studies have proven the effectiveness of phosphate solubilizing bacteria (PSB) in improving crop yield due to improved P levels in the soil (Khourchi et al., 2022b). Kohler et al. (2009) found that *Pseudomonas mendocina* enhanced the salt tolerance of lettuce plants resulting in a reduction of catalase activity with an increase in shoot dry weight and proline concentration in leaves. Furthermore, in a recent study, Belouchrani et al. (2020) showed that phosphorus supplies improved sorghum tolerance to soil salinity which is observed by an increase in morphological parameters, nitrogen and phosphorus uptake, and proline accumulation. In a growing hydroponic condition, it has been observed in salt-stressed tomato plant that increasing the phosphorus amounts in the solution improve root length and root surface area (Loudari et al., 2020). Also, in barley plants, increasing plant phosphorus in nutrient solution enhances salt tolerance by reducing sodium and increasing potassium (K) concentrations in the shoot (Chen et al., 2007). In pepper and cucumber plants grown under salinity, the supply of KH_2_PO_4_ mitigated the harmful effects of salinity on fruit yield and plant biomass and restored the K and P in leaves and roots (Kaya et al., 2013). Shibli et al. (2001) concluded that P plays a pivotal role in understanding the physiological response to salt stress in different plant species. At the microculture level of African violet (*Saintpaulia ionantha*), P supply restored nutrient uptake (Shibli et al., 2001). This positive effect was also tested by the foliar application of P in wheat plants (Khan et al., 2013) and common bean plants (Rady et al., 2018) grown under salinity, revealed by the increase in the total performances of plants. Hence, while the interaction between P and salt stress positively affects plant growth and yield, there is an urgent need to concentrate also on the reasonable application of more efficient P sources in order to cope with the limited P availability and improve plant productivity mainly in salt-affected soil. Polyphosphates (PolyP) have been applied in agriculture and are renowned for releasing available P to plants in agricultural soil slowly and continuously (Kulakovskaya et al., 2012). These characteristics make PolyP a sustainable source of P to satisfy plant requirements and prevent phosphorus losses in soils over time (Khourchi et al., 2022a). Furthermore, it has also been found that PolyP fertilizers differ from orthophosphates (OrthoP) by their capacity to chelate certain micronutrients such as manganese, iron, and zinc (Wang et al., 2019; Gao et al., 2020). Compared to OrthoP, the plant responses to PolyP application under saline conditions are not commonly studied. In our study, we posed the question if soluble P-fertilizers allow wheat plants to counter the adverse effect of salt stress. Hence, we hypothesize that using contrasting forms of P-fertilizers at various P doses could have a positive effect on durum wheat growth under moderate salt stress. Three soluble fertilizers were used: Two Orthophosphates and one polyphosphate were applied at different P levels. Afterwards, wheat plant growth, physiological parameters (chlorophyll content index, stomatal conductance, chlorophyll a fluorescence), and mineral uptake were assessed.

## 2 Material and methods

### 2.1 Plant material, fertilisation, and experimental design

The experiment was installed in open field conditions at the Experimental Farm of Mohammed VI Polytechnic University (UM6P), Benguerir, Morocco. During the growth season, the temperatures in Benguerir ranged from 0°C (minimum) and 45°C (maximum), with an average of 19°C. The mean light intensity per day was around PAR 280 µmol m^−^2 s ^−1^. The cumulative rainfall during December, October, May, March, and January was 99 mm. A representative soil sample from a 20 cm layer of agricultural land (Rass El Ain- Morocco) was collected and analysed before the experiment to refine the treatments (pH, Electrical Conductivity (EC), texture, assimilable Phosphorus (P), Total Nitrogen (NT), Organic matter (OM), Na2O, Potassium (K), CaCO3, micronutrients.). For every analysis, we have undertaken three repetitions. EC was determined with Conductivity-meter in dS m^−1^. The pH of the soil was determined in deionized water. Phosphorus in percentage was revealed by OLSEN Method, Organic matter (OM) in, OM % = Organic Carbon (Corg) % × 1.72. Cation exchange capacity (CEC) was determined by the percolation method with ammonium acetate 1 N. The results of soil analysis are reported in **Table 1**. The soil has the same properties as most soils of the R’hamna region- Morocco but was mainly moderately deficient in assimilable P (P_2_O_5 =_ 30.33 ppm). The soil was air-dried and sieved (8 mm). Each pot was preliminarily filled with a thin layer of gravel (1 cm). The deficit nutrients were added according to the method suggested by COMIFER (French Committee for the Study and Development of Reasoned Fertilization). Basal amendment consists of three different doses of phosphorus (0, 30 and 45) for each NPK soluble fertilizer (Ortho-A, Poly-B and Ortho-B). The OrthoP fertilizers used in the experiment are phosphoric acid-based fertilizers with potassium (Ortho-A) or Nitrogen (Ortho-B) containing 52% and 62% of P_2_O_5_, respectively, with 100% OrthoP for each one. Poly-B fertilizer is a linear PolyP with a short chain which contains 47% P_2_O_5_ with 100% PolyP in form of tripolyphosphates. According to wheat requirements and to the amount of nitrogen and potassium in the selected soil and fertilizers, NH₄NO₃ (33.5% N) and potassium sulphate (51% K_2_O) were applied to equalize N and K amounts for all treatments. The quantities were adjusted also for controls. A control combination consists of negative control (C-): unfertilized plants without salt application, and positive control (C+): salt-stressed and unfertilized plants.

**Table 1:** Physicochemical properties of the experimental soil.

Soil parameters		
**Soil Texture**	Clay (%)	15
	Slit (%)	26
	Sand (%)	58
**EC ext1/5 (dS/m)**		1,587
**pH_water**		7,893
**P2O5 (ppm)**		30,33
**K2O (ppm)**		228,3
**N-NO3 (mg/Kg)**		54,017
**N-NH4 (mg/Kg)**		7,893
**MO (%)**		3,11
**C_org_ (%)**		1,806
**CaCo3 total (%)**		2,490
**C.E.C (meq/100g)**		12
**Na2O (ppm)**		1546,66
**MgO (ppm)**		624
**CaO (ppm)**		6472
**Cu (ppm)**		0,71
**Mn (ppm)**		11,04
**Fe (ppm)**		6,26
**Zn (ppm)**		0,62

A Moroccan variety of durum wheat (*Triticum durum*) was used. Karim cultivar is one of the most cultivated varieties in Morocco, known for its adaptation zone (bour and irrigated lands), its precocity, medium straw production, and tolerance to rust and *Septoria*. Ten dry, healthy, and uniform size seeds were sown into polyethene pots (24 cm in diameter and 35 cm in length) containing 10 kg of dried soil per pot, and only six seedlings (same size and appearance) were kept after plant emergence. The experiment was conducted in a completely randomized design with ten replicates per treatment. During the experiment, the plants were watered with rap water when soil moisture content had fallen to 60% of its initial value. Initial electrical conductivity of soil was EC= 1,587 dS/m (**Table 1**). 

The salinity treatment was applied by adding saline water (with definite EC) after seedlings establishment, which is usually two weeks after sowing (WAS). The salinity level was gradually increased in order to achieve moderate saline conditions (EC= 3.003 dS/m). Moisture and EC were measured before and after each irrigation using the HH2 WET sensor (Delta-T devices). During the wheat growth, the measurements were taken every two (WAS), starting from 6 WAS. The samples of plants and rhizosphere soils were taken at 12 WAS, which corresponds to Z68 – Z71 of Zadok’s scale (the heading stage).

### 2.2 Chlorophyll content index

Chlorophyll Content Index (CCI) was estimated by using a non-destructive portable chlorophyll meter (CL-O1, Hansatech instruments). This parameter was measured from the middle part of the fully mature and expanded functional leaves after 1 min kept in dark. CCI was measured in all treated plants at 6, 8, 10 and 12 weeks after sowing. At each treatment, the CCI was measured at least on 12 independent leaves.

### 2.3 Stomatal conductance

Stomatal conductance (SC) was measured by a leaf porometer (SC-1 Leaf porometer Decagon Devices, Inc.) in the morning and was measured from the middle part of the fully mature and expanded functional leaves in all treated plants at 6, 8, 10 and 12 weeks after sowing. At least 5 independent measurements were taken.

### 2.4 Chlorophyll a fluorescence and total photosynthetic performance

Chlorophyll *a* fluorescence of wheat leaves held in dark for 15 minutes was measured by using a handheld fluorometer (Handy PEA+, Hansatech instruments). For each treatment, at least 15 measurements were made from the middle part of the fully mature and expanded functional leaves, and each measurement consisted of 1s single and strong light pulse (3000 μmol s^-1^ m^-2^), this light is provided by an array of six light-emitting diodes (peak 650 nm). The OJIP fluorescence curve is a typical curve of chlorophyll fluorescence with the three transition phases (OJ, JI and IP). The O–J phase indicates a photochemical phase, and the J–I–P phase indicates a thermal phase (Stirbet, 2012). This OJIP transient reflects diverse reduction processes of the electron transport chain (Strasser et al., 2004; Stirbet, 2012). The photochemical phase O–J is reported to be deeply light-dependent (Schansker et al., 2006) and informs connectivity between PSII reaction centres. The thermal phase, J to P rise, indicates a reduction of the rest of the electron transport chain (Kalaji et al., 2017).

The fluorescence parameter PI_total_ was calculated from the fluorescence transient measured during the 1^st^ second of illumination. PI_total_ (1) is estimated to be a product of the PI_ABS_ (photosynthetic performance index) (2) and the probability that an electron (e-) can move from the reduced intersystem electron acceptors to the PSI end-electron-acceptors (3) (Tsimilli-Michael and Strasser, 2008):

*PI*_*total*_= *PI*_*ABS*_.*δ*_*Ro*_/(1−*δ*_*Ro*_)(1)


(2) $$PI_{ABS}=[RC/ABS]\times [\varphi_{Po}/(1-\varphi_{Po})]\times [\psi_{o}/(1-\psi_{o})]$$

With:

ABS/RC: Specific absorption flux per reaction centre (RC)

φ_Po_: Quantum yield of electron transport (at t = 0), φ_Po_ = (1−F_O_/F_M_)

ϕ_o_: Probability (at t = 0) that a trapped exciton moves an electron into the electron transport chain beyond *Q*_*A*_^−^, ψ_o_ = 1 – V_J_

δ_Ro_ indicates the efficiency with which an electron can move from the reduced intersystem electron acceptors to the PSI end electron acceptors, and can be expressed as:


(3) $$\delta_{Ro}=\;\left(1-V_{I}\right)/\left(1-V_{J}\right)$$

V_t_ (4) is described as the relative variable Chl *a* fluorescence at time t. It corresponds to:


(4) $$Vt\;=\left(F_{t}–\;F_{o}\right)/\left(F_{M}-\;F_{o}\right)$$

This equation can be identified as a measure of the fraction of the primary quinone electron acceptor of PSII in its reduced state [Q_A_^-^ /Q_A (total)_]. ΔVt (5) could indicate additional information and bands that might be hidden in the kinetic curves of Chl a fluorescence OJIP (Chen et al., 2016). It was calculated as the difference between Vt values obtained by plants at the different P doses (0, 30 and 45 ppm P minus the respective values of unfertilized plants without salt stress (negative control):


(5) $$\Delta Vt\;=\;Vt\;\left(P\right)\;-Vt\;\left(P-P_{negative\;control}\right)$$

### 2.5 Biomass

Plants were separated into shoots and roots, washed and dried at 75°C in an oven until the root and shoot dry weights stabilized.

Root and shoot Tissue Water Content (TWC) was calculated using the following formula (6):


(6) TWC= (FW−DW)/DW

With:

FW: Fresh weight, DW: Dry weight

### 2.6 Nutrient analysis

Elemental concentrations of P, K, and Na were analyzed on a dry-weight basis using Inductively Coupled Plasma Optical Emission Spectrometry (Agilent 5110 ICP-OES, USA).

### 2.7 Statistical analysis

Statistical analysis was performed using one-way ANOVA (for P< 0.05) and SPSS data processing software (SPSS 20.0), considering three independent replicates per treatment. Based on the ANOVA results, and for a 95% confutation level, a GT2 of the Hochberg test for the comparison of means was performed, to reveal the significant differences between treatments. Pearson’s Correlation coefficients r were calculated to determine the association between dry weight yield of shoot and root and their mineral content.

## 3 Results

### 3.1 Chlorophyll content index

**Figure 1:**
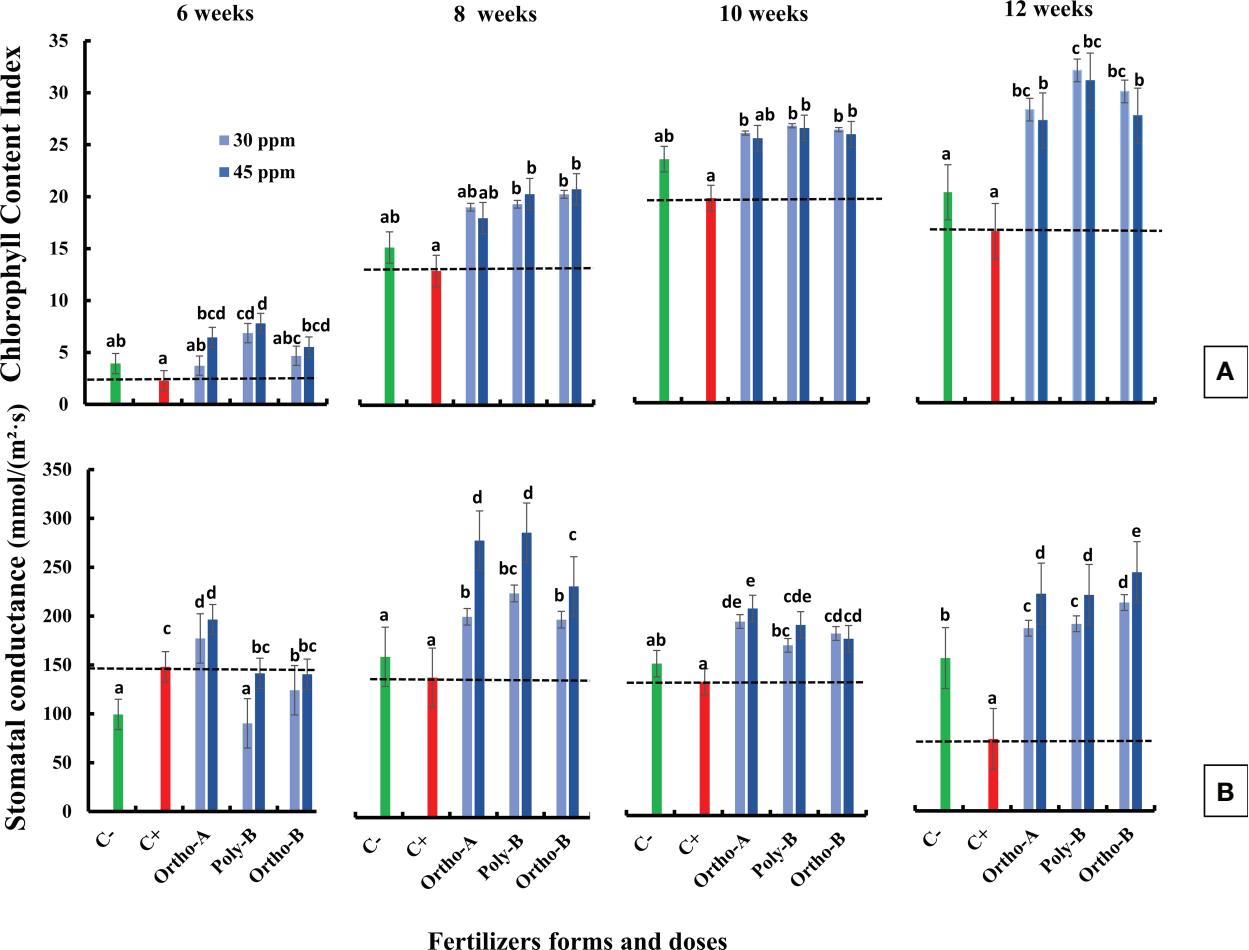
The combined effect of P-fertilizer forms (Ortho-A, Poly-B and Ortho-B) and doses (0, 30 and 45 ppm) on Chlorophyll content index (CCI) **(A)** and Stomatal conductance (SC) **(B)** of wheat plants grown under salt stress conditions, measured at 6, 8, 10 and 12 WAS. C-: unfertilized plants without salt application, C+ salt-stressed and unfertilized plants. Statistical analysis was performed using one-way ANOVA and SPSS data processing software. GT2 of the Hochberg test was used for the comparison of means. Treatments having the same letters are not significantly different at the 5% level.

Chlorophyll Content Index (CCI) measured in salt-stressed and unfertilized plants (C+) showed a reduction compared to measured CCI in unfertilized plants without salinity stress (C-) (**Figure 1A**). For instance, growth at 12 weeks after sowing, CCI reduced by 22.6% in C+ compared to C- plants. However, fertilized plants showed an increase in CCI compared to control plants (C+ and C-). After 12 weeks of growth and with a dose of 30 ppm of P, CCI increased by 93%, 81%, and 71% in plants fertilized by Poly-B, Ortho-B and Ortho-A, respectively compared to C+ (**Figure 1A**). The different doses of P in the different fertilizers did not show a significant effect on CCI. The difference between fertilizer forms was significant mainly for Poly-B which increased by 17.42% at 30 ppm f P compared to Ortho-A and Ortho-B at 45 ppm of P.

### 3.2 Stomatal conductance

Stomatal conductance (SC) decreased in salt-stressed and unfertilized plants (C+) compared to unfertilized plants without salinity stress (C-) except at the beginning of growth, 6 weeks after sowing (**Figure 1B**). This decrease was significant in plants grown 12 weeks after sowing. However, fertilized plants showed a significant increase in SC compared to C- and C+ plants except for plants grown 6 weeks after sowing. Furthermore, P dose in different soluble fertilizers showed a significant effect on SC while the fertilizers forms did not affect this physiological parameter. Indeed, compared to C+, Poly-B and Ortho-A showed similar results in SC with an increase of 157% and 217% at 30 and 45 ppm of P, respectively. The highest significant value of SC was obtained with Ortho-B fertilizer at 45 ppm with an increase of 232% and 56% compared to C+ and C- plants, respectively.

### 3.3 Chlorophyll a fluorescence and photosynthetic performance index

**Figure 2:**
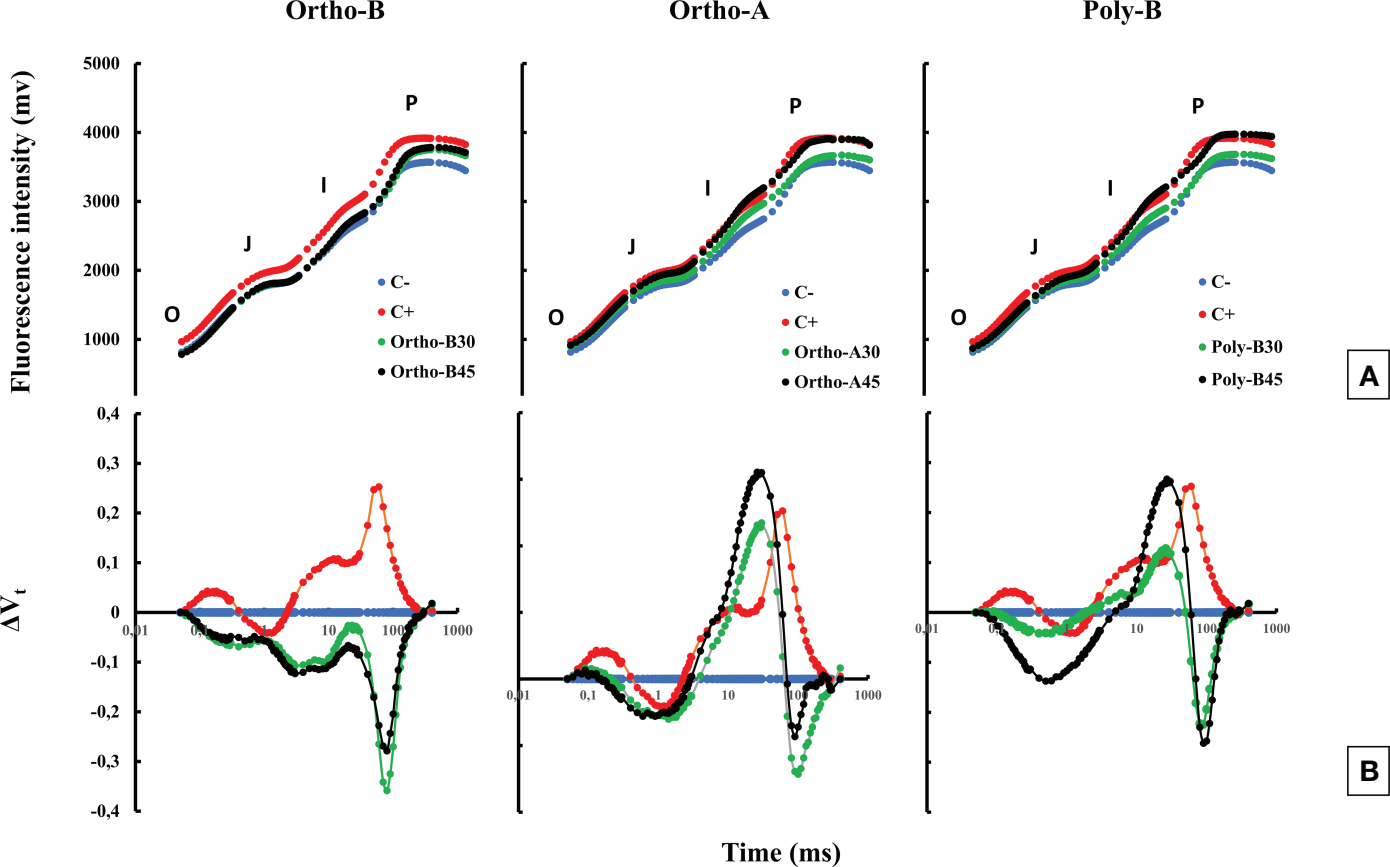
The combined effect of P-fertilizer forms (Ortho-A, Poly-B and Ortho-B) and doses (0, 30 and 45 ppm) on OJIP curves **(A)** and ΔVt fluorescence parameter **(B)** of wheat plants grown under salt stress conditions at 12 WAS. C-: unfertilized plants without salt application, C+ salt-stressed and unfertilized plants.

**Figure 3:**
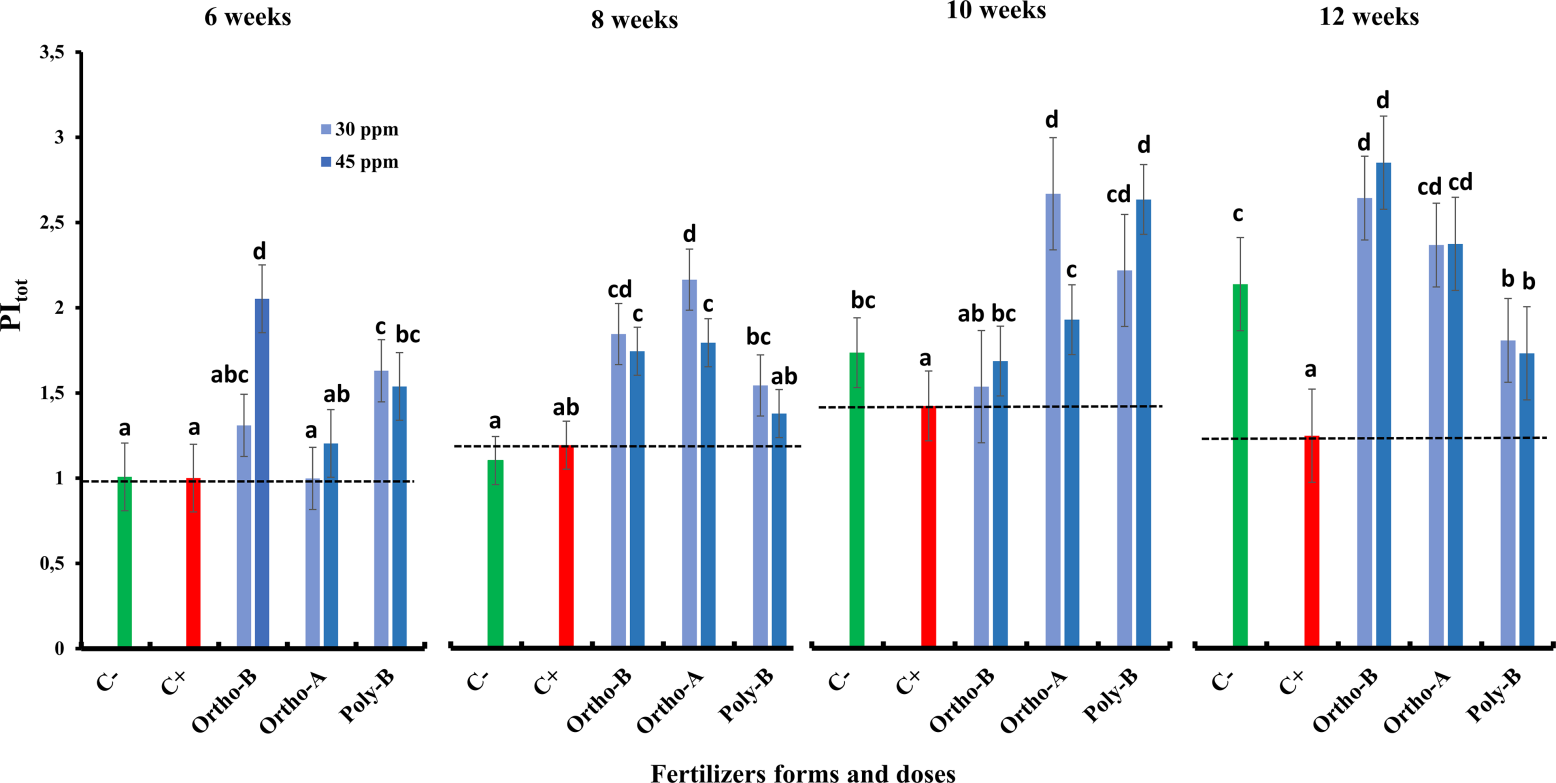
The combined effect of P-fertilizer forms (Ortho-A, Poly-B and Ortho-B) and doses (0, 30 and 45 ppm) on Photosynthetic Performance Index (PI _tot)_ of wheat plants grown under salt stress conditions, measured at 6, 8, 10 and 12 WAS. C-: unfertilized plants without salt application, C+ salt-stressed and unfertilized plants. Statistical analysis was performed using one-way ANOVA and SPSS data processing software. GT2 of the Hochberg test was used for the comparison of means. Treatments having the same letters are not significantly different at the 5% level.

**Figure 2A** shows no visual difference in the effect of salinity on the fluorescence curve; however, a difference in fluorescence yield in the J-I-P phase was observed. The subtraction of the different curves from the curve measured in the negative control (C-) plants (ΔVt) showed the presence of two bands (**Figure 2B**), the first in the J-I phase and the second during the I-P phase. In salt-stressed and unfertilized plants (C+), measurements showed only a single positive band in the J-I phase and another band was also observed in the O-J phase with a peak of around 300 µs. The fully mature leaves of fertilized wheat plants showed a significant increase in the photosynthetic performance index PI_tot,_ compared to negative (C-) and positive control (C+) plants (**Figure 3**). Furthermore, the P dose and fertilizers forms showed a significant effect on PI_tot_.

Compared to C+, the PI_tot_ was increased by 128.5%, 90.2% and 38.8% for Ortho-B, Ortho-A and Poly B, respectively. For the same fertilizer, the dose did not affect the PI_tot_. 

### 3.4 Biomass and tissue water content

**Figure 4:**
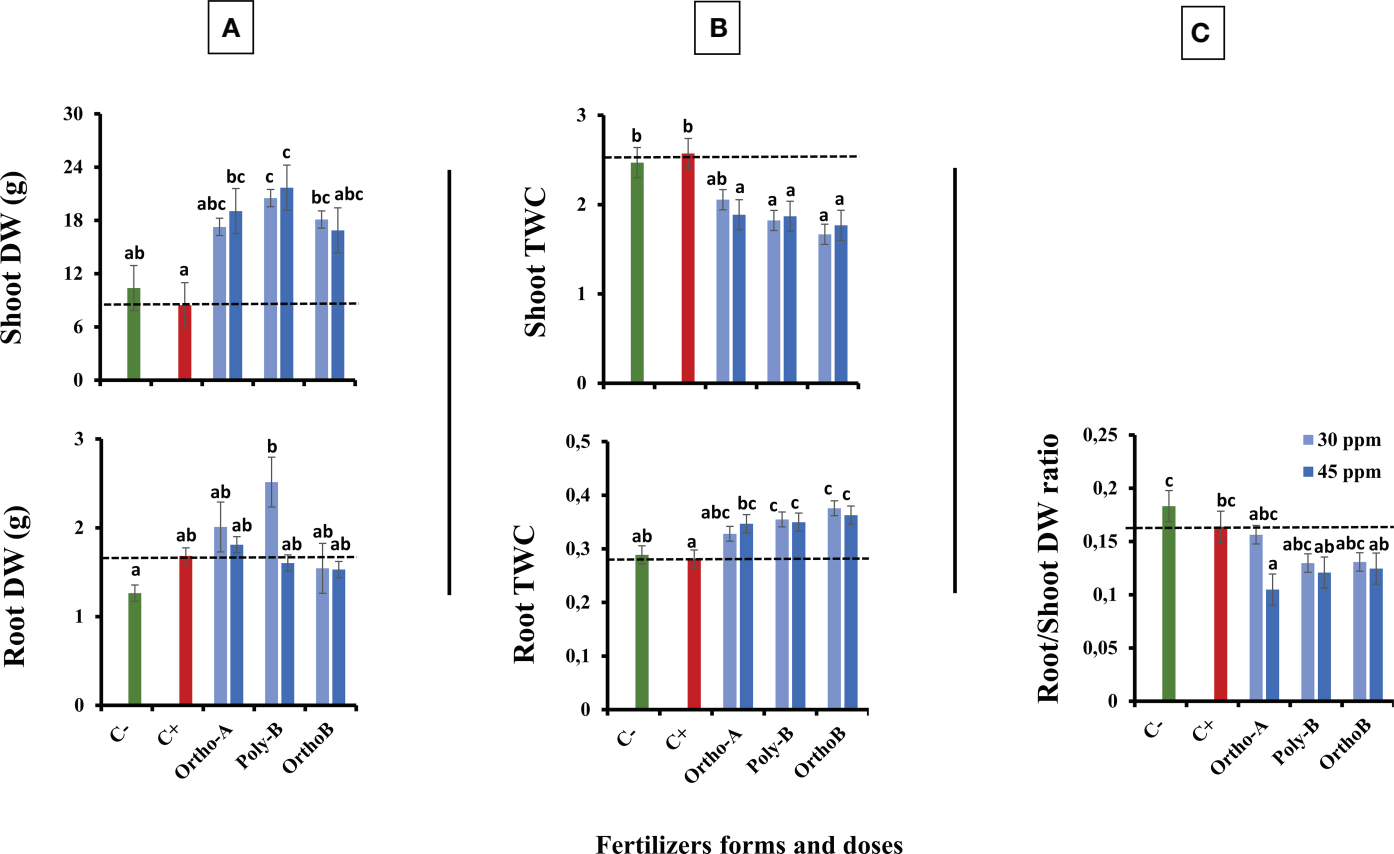
The combined effect of P-fertilizer forms (Ortho-A, Poly-B and Ortho-B) and doses (0, 30 and 45 ppm) on Shoot and Root dry weight (DW) **(A)**, Shoot and Root Tissue water content (TWC) **(B)** and Root/Shoot DW ratio **(C)** of wheat plants grown under salt stress conditions, measured at 12 WAS. C-: unfertilized plants without salt application, C+ salt-stressed and unfertilized plants. Statistical analysis was performed using one-way ANOVA and SPSS data processing software. GT2 of the Hochberg test was used for the comparison of means. Treatments having the same letters are not significantly different at the 5% level.

**Figure 4A** shows an increase in the dry weight (DW) of the shoot in fertilized plants compared to unfertilized plants exposed to salinity (C+) or not (C-). The source of fertilizers has a significant effect but depends on the dose of P. The increase in shoot DW was significant mainly with Poly-B fertilizer (156.8%) for both doses followed by Ortho-A (125.6%) and Ortho-B (114.2%) at 45 and 30 ppm of P, respectively in comparison with C+ plants. However, the dry weight of the roots depends both on the dose and the form of the soluble fertilizers. Furthermore, Poly-B fertilizer showed the best performance at 30 ppm of P with an increase of 49.4% and 98.9% in root DW compared to C+ and C- respectively, while other P-treatments did not show a significant difference with the C+. In addition, Root Tissue Water Content (TWC) (**Figure 4B**) significantly decreased in unfertilized plants under saline conditions (C+) or not (C-) compared to salt-stressed and fertilized plants. The root TWC increased by 33.7% compared to C+ for Ortho-B and Poly B with a similar response for both doses, followed by Ortho-A (23.5% and 16.73% for 45 and 30 ppm of P, respectively). However, our results showed that the shoot TWC in unfertilized plants has not been reduced under salinity, whereas it has been significantly decreased for other P-treatments. This response was not strongly influenced by forms or doses of P-fertilizers (**Figure 4B**). After 12 weeks after sowing, the ratio of the DW of roots to the DW of shoots decreased in the salt-stressed and fertilized plants compared to unfertilized plants (C+ and C-)(**Figure 4C**). At 45 ppm of P, Ortho-A showed a significant decrease in this ratio (-56%) compared to 30 ppm of P and C+ while the Ortho-B and Poly-B treatments did not show significant differences between each other. 

### 3.5 Mineral analysis

#### 3.5.1 Root and shoot mineral content

**Figure 5:**
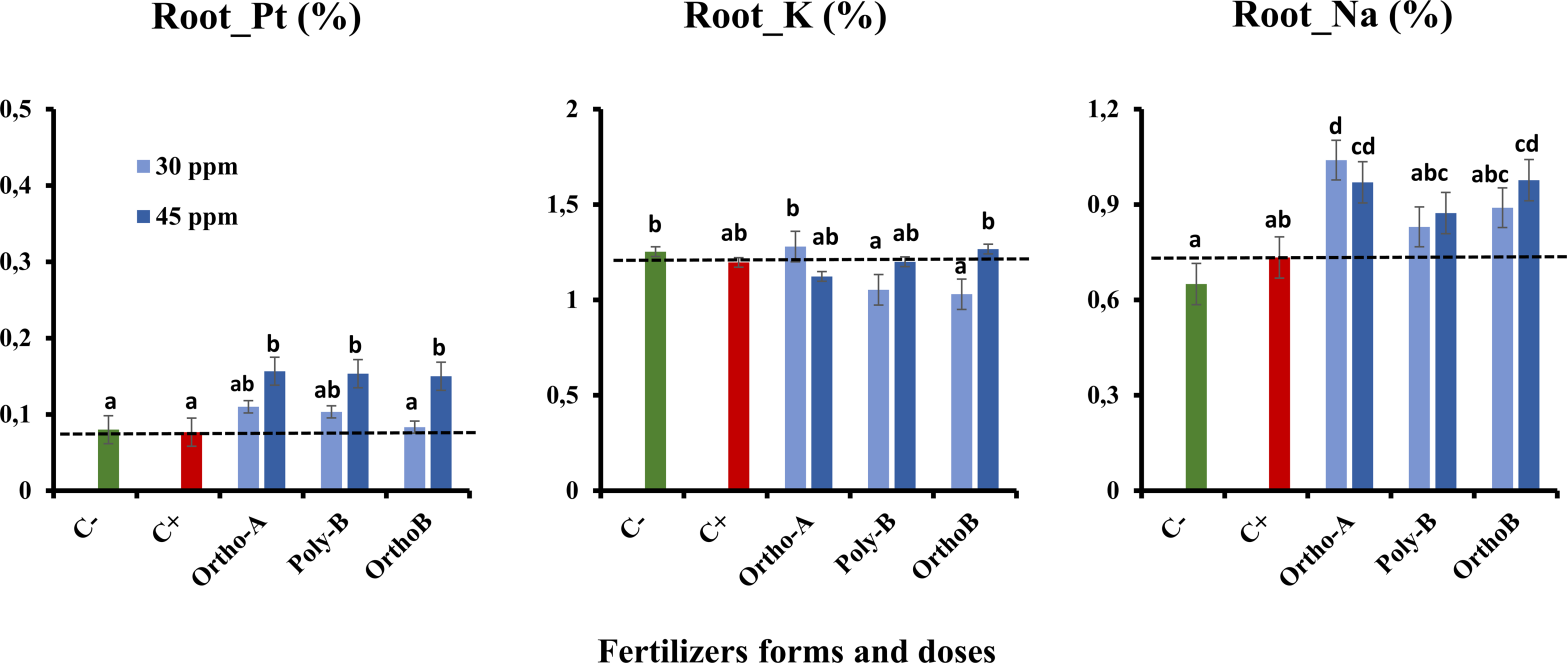
The combined effect of P-fertilizer forms (Ortho-A, Poly-B and Ortho-B) and doses (0, 30 and 45 ppm) on total phosphorus (Pt), Potassium (K) and Sodium (Na) in roots of wheat plants grown under salt stress conditions, measured at 12 WAS. C-: unfertilized plants without salt application, C+ salt-stressed and unfertilized plants. Statistical analysis was performed using one-way ANOVA and SPSS data processing software. GT2 of the Hochberg test was used for the comparison of means. Treatments having the same letters are not significantly different at the 5% level.

**Figure 6:**
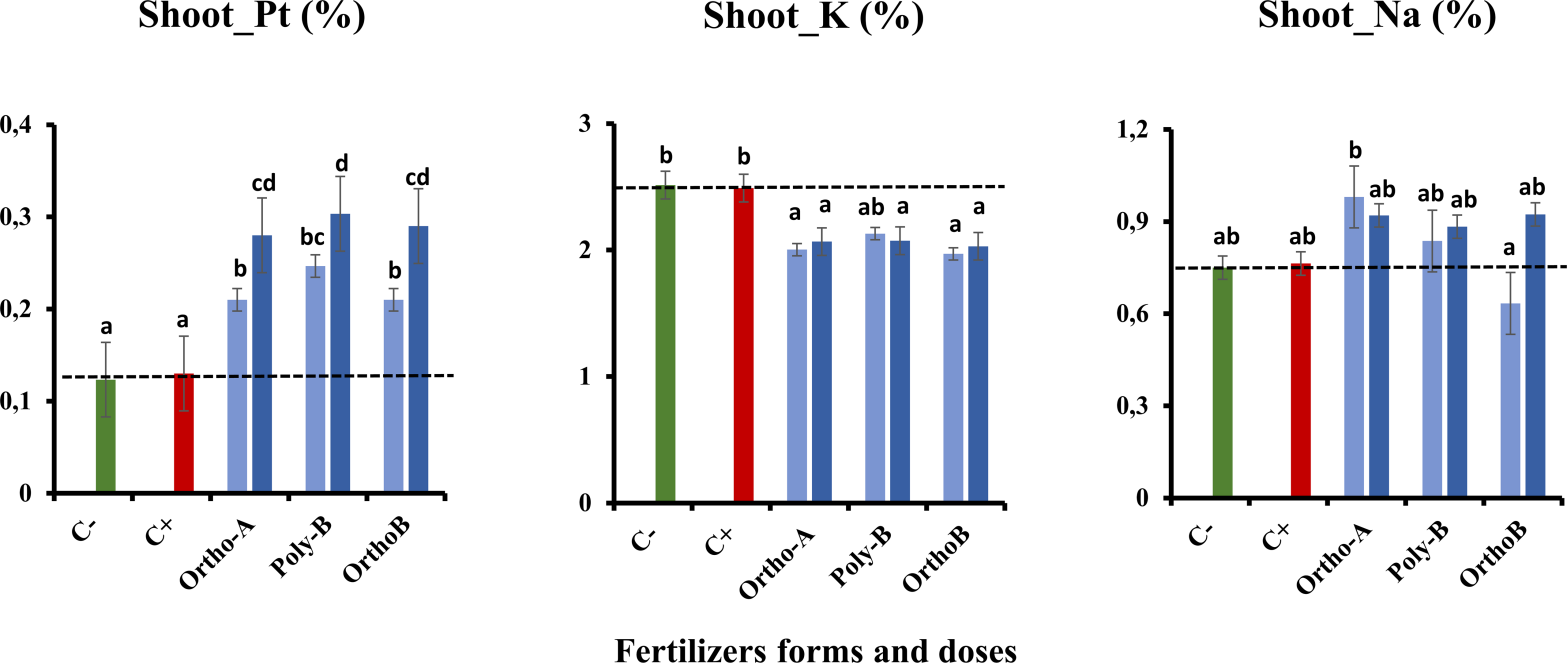
The combined effect of P-fertilizer forms (Ortho-A, Poly-B and Ortho-B) and doses (0, 30 and 45 ppm) on total phosphorus (Pt), Potassium (K) and Sodium (Na) in shoots of Wheat plants grown under salt stress conditions, measured at 12 WAS. C-: unfertilized plants without salt application, C+ salt-stressed and unfertilized plants. Statistical analysis was performed using one-way ANOVA and SPSS data processing software. GT2 of the Hochberg test was used for the comparison of means. Treatments having the same letters are not significantly different at the 5% level.

**Figures 5**, **6** show an increase in the total P (Pt) content in the root and shoot of fertilized plants compared to unfertilized plants exposed to salinity (C+) or not (C-). The form of fertilizers has a significant effect but depends on the P dose. At 45 ppm of P, P fertilizers showed similar results with an increase of 104% in Pt root content of salt-stressed plants in comparison with C+ (**Figure 5**). However, Ortho-B did not show any difference with the C+ at 30 ppm of P. The same tendency was observed for Pt shoot content, where the dose 45 ppm of P showed a significant rise in shoot-Pt for all P fertilizers compared to the 30 ppm dose (**Figure 6**). Compared to unfertilized plants (C- and C+), OrthoP fertilizers (Ortho-A and Ortho-B) showed similar results for both doses with an increase of 62% and 115% at 30 and 45 ppm of P, respectively. The Pt shoot content was significantly improved using Poly-B fertilizer compared to C+ and C- and showed the highest significant Pt accumulation in shoots estimated by 84% and 131% at 30 and 45 ppm of P, respectively (**Figure 6**). The response was, therefore, dose/form dependent. However, the K shoot content decreased significantly for all fertilized plants compared to unfertilized ones (-20%) (**Figure 6**). Accordingly, there is no significant difference between P-treatments and controls (C+ and C-) in the amount of K in root except for Ortho-B and Poly-B at 30 ppm of P which showed the lowest value of K accumulation (**Figure 5**). As unexpected results, the Na root content increased in the root and shoot of fertilized plants compared to unfertilized ones under salinity (C+). The effect was more relevant using Ortho-A at 30 ppm of P with an increase of 28% and 42% in Na content in shoots and roots, respectively (**Figures 5**, **6**).

#### 3.5.2 Correlation matrix

Pearson’s correlation coefficients among plant dry weight (shoot and root dry weight) and different nutrients in the shoot and root of wheat salt-stressed plants cultivated with different forms and doses of soluble P-fertilizers were shown in **Table 2**. At 12 WAS, there was no significant correlation between shoot and root DW, but a positive significant correlation was observed between shoot-Pt content (P ≤ 0.01) and Shoot DW (r = 0,770**), root-Na (r = 0,587**) and root-Pt content (r = 0,742**). However, there was a negative significant correlation between shoot-K content (P ≤ 0.01) and Shoot DW (r = - 0,637**), root-Na (r = - 0,686**), Pt-shoot (r = - 0,653**) and Pt-root content (r = - 0,514*) (P ≤ 0.05). The latter shows a positive significant association (P ≤ 0.05) with the shoot DW (r = 0,491*), Na-shoot (r = 0,486*). The Pt-root content was also significantly correlated (P ≤ 0.01) to shoot-Pt content (r = 0,742**) and Na-root content (r = 0,554**).

**Table 2:** Pearson’s correlation coefficients among plant dry weight (shoot and root dry weight) and measured nutrients in the shoot and root of wheat plants grown with different forms and doses of soluble P-fertilizers under salinity at 12 WAS (n = 24).

Pearson’s correlation coefficients								
Trait	Shoot_Pt	Shoot_K	Shoot_Na	SDW	Root_Pt	Root_K	Root_Na	RDW
**Shoot_Pt**	1,00	-0,653**	0,32	0,770**	0,742**	-0,11	0,587**	0,21
**Shoot_K**		1,00	-0,10	-0,637**	-0,514*	0,10	-0,686**	-0,21
**Shoot_Na**			1,00	-0,10	0,486*	0,12	0,35	0,23
**SDW**				1,00	0,491*	-0,19	0,39	0,23
**Root_Pt**					1,00	0,27	0,554**	-0,05
**Root_K**						1,00	0,26	-0,38
**Root_Na**							1,00	0,12
**RDW**								1,00

** and *: significant at 0.01 and 0.05 levels, respectively; Pt, total phosphorus; K, potassium; Na, sodium; SDW, shoot dry weight, RDW, Root dry weight, WAS, Week after sowing.

## 4 Discussion

Salt stress limits wheat growth and development by inducing a series of physiological dysfunctions in different organs such as leaves, shoots, and roots. However, soluble fertilizer forms and P doses enhanced plant responses and countered the negative effect of this stress.

### 4.1 Salt-stressed and unfertilized wheat plants’ responses

The reduction of chlorophyll content in salt-stressed leaves of wheat observed in our study was reported in many studies focused on the effect of salinity on plants (Ashraf and Harris, 2013; Sharma et al., 2020). A significant decrease in CCI was observed at 6, 8,10 and 12 WAS, which reached -23%. In C+ plants compared to C- (**Figure 1A**). This reduction has been associated with an increase in chlorophyllase activity which is an enzyme degrading the chlorophyll (Shoukat et al., 2019) and with the instability of pigment-protein complexes (Renger et al., 2011). Also, photoinhibition and reactive oxygen species (ROS) formation during salt stress could cause a decrease in chlorophyll content (Hasanuzzaman et al., 2021; Muhammad et al., 2021). Additionally, the decline of stomatal conductance was significantly observed in unfertilized and salt-stressed wheat plants (C+) compared to C-. The difference was significant at 12 WAS with a reduction of -113% in C+ compared to C- plants (**Figure 1B**). This is consistent with previous observations on the effect of salt stress on plants (Lotfi et al., 2020; Behdad et al., 2021). As was reported, salt stress provokes osmotic stress in the root, and then the limited water absorption affects the aperture of stomata to preserve water in plant tissues and decrease water loss *via* transpiration (Zribi et al., 2021). In such situations, plants usually adopt defensive strategies by the increase of water use efficiency (WUE), control of the net CO2 and the rate of transpiration in leaves (Muhammad et al., 2021). However, under severe salt stress conditions, the mesophyll cell dehydration allows the use of available CO2, which significantly inhibits photosynthesis metabolic processes, leading to a decrease in water use efficiency and hydraulic conductivity of root (Din et al., 2011). Indeed, stomatal conductance plays an essential role in water balance regulation and stomatal closure has also a direct effect on plant growth by reducing cell expansion and plant development causing a decrease in biomass and plant productivity (Nemeskéri et al., 2019). Fahad et al. (2015) reported that the major effects of moderate salt stress on growth could be attributed to a major investment of energy in defence mechanisms rather than in biomass production. Accordingly, root and shoot dry weights (DW) decrease toward salt stress (Fig 4. A) which has also been reported in previous studies (Yan and Marschner, 2012; Muhammad et al., 2021). This reduction was more relevant in shoot DW than root DW in comparison with C- and fertilized plants. Eker et al. (2006) reported that root DW was less affected by salinity than shoot DW for two varieties of hybrid maize. These findings indicate that shoot growth could be a more useful parameter than root growth for assessing the salinity tolerance of plants. We assume that the decrease in dry biomass was resulting in the reduction of chlorophyll content and stomata closure (Nemeskéri et al., 2019). This positive association between photosynthetic capacity and biomass production has been confirmed under salinity for maize plants (Hessini et al., 2019), quinoa (Manaa et al., 2019) and pepper (Altuntas et al., 2018).In the last decades, the root/shoot ratio was adopted for assessing plant growth and was considered a sensitive growth parameter and indicator in plant stress physiology (Rahimi et al., 2021). To minimize the negative effect of salt stress, the plant developed phenotypic plasticity (Rewald et al., 2012). Contrary to what has been reported in previous studies, that the root/shoot ratio increased in stress conditions (Khorshid et al., 2018; Rahimi et al., 2021), in our investigation, this ratio decreased for all P treatments. This reduction was more relevant at 45 ppm of P for Ortho-A followed by Poly-B and Ortho-B which were similar for both doses of P. Thus, biomass was more allocated in shoots than in roots. Contradictory reports exist regarding the influence of salinity and P deficiency on the root/shoot biomass ratio. Low P availability has been shown to increase the allocation of dry matter to roots while suppressing shoot growth, resulting in increased root/shoot ratios (Kim and Li, 2016). This ratio has been reported to be affected (increase or decrease) in different plants like tomato and petunia (Kim et al., 2008), common bean (Lynch and Brown, 2006) or unaffected (Broschat and Klock-Moore, 2000). Biomass allocation to root or shoot depends on the salt degree, time and duration of exposure, plant species, and developmental stage (Shabala and Munns, 2017).

The salt-stress effects on photosynthesis range from the limitation of CO_2_ diffusion into the chloroplast, through limiting stomatal opening, which is regulated by hormones produced in shoot and root, and on the CO_2_ mesophyll transport, up to major modifications in the photochemistry of leaves and C metabolism, or they may induce oxidative stress. This appears as a secondary effect (Chaves et al., 2011), which can seriously alter the photosynthetic machinery of leaves (Sharma et al., 2020; Muhammad et al., 2021). Moreover, Dekker and Boekema (2005) reported that the key functional chloroplast protein complexes, implicated in harvesting light energy (PSI, PSII, ATP-synthase and Cytb6f), are affected in salt-stressed plants. The changes in the oxygen-evolving complex (OEC) and proteins of the PSII reaction centre are recognized to enable PSII to deal with saline environments (Duarte et al., 2013; Oukarroum et al., 2015). Furthermore, it has been previously demonstrated that OJIP transient shape changes under different abiotic stresses including salt stress (Sarkar and Ray, 2016). This change differs depending on the severity and duration of stress. In our study, the thermal phase J-I-P seems to be affected by salinity (**Figure 2A**). The difference between fertilized and C- plants was determined in salt-stressed wheat and showed a positive band with a pic at 300 us (**Figure 2B**). The appearance of this band named K-band reflects a restriction on the donor side of PSII (Strasser et al., 2004; Oukarroum et al., 2007). This K-band can be seen in the fluorescence rise of, e.g., plants under heat and drought stress (Brestic et al., 2012). We found that the salinity stress induced a reduction in the photosynthetic performance index PI_tot_ (**Figure 3**). The estimated performance index reflects the photosynthetic performance up to the reduction of PSI end e- acceptors. The highest significant difference between C+ and C- plants (−71%) was observed at 12 WAS which suggests an additive effect of salinity and P deficiency over time. This decrease in PI_tot_ indicates that the plant vitality was inhibited to a certain degree under our salinity and P deficiency conditions.

Among the negative consequences of salt stress on plants is ROS formation. It has been well reported that ROS can damage cellular components and disturb many physiological mechanisms (Kumar et al., 2017; Hasanuzzaman et al., 2021). Moreover, ROS acts also as signal transduction in cells to reduce this effect in stressed plants (Kumar et al., 2017).

### 4.2 Salt-stressed and fertilized wheat plants’ responses

Major effects of moderate salt stress on growth could be attributed to a major investment of energy on defence mechanisms rather than on biomass production (Fahad et al., 2015) or due to the reduced water uptake which leads to a reduction in toxic ion assimilation (Shabala and Munns, 2017; Zhao et al., 2020). Furthermore, when P nutrition was sufficient, growth reductions and visual symptoms of salt toxicity were minimized and were more accentuated by P deficiency (Mohamed et al., 2021; Zribi et al., 2021). Supply soluble fertilizers enhance wheat growth and improve salt tolerance as observed in all studied parameters. This positive role has been previously observed in other plant species exposed to salt stress and supplied by different P doses (Khan et al., 2013; Bargaz et al., 2016; Rady et al., 2018; Bouras et al., 2021; Mohamed et al., 2021; Zribi et al., 2021; Bouras et al., 2022). We assume that adding P to plants grown under salt stress could mitigate the negative effects caused on different plant organ development. Indeed, it has been shown that phosphorus is an important factor in the growth of shoots and roots, and low phosphorus uptake under salinity may reduce biomass development (Demiral, 2017; Khan et al., 2018). In the present work, shoot and root dry weights significantly declined in unfertilized plants grown under salinity (C+) compared to fertilized ones (**Figure 4A**). These findings are in line with previous reports (Parvez et al., 2020; Zribi et al., 2021). This reduction might be a plant survival strategy associated with carbon (C) assimilation failure (Shoukat et al., 2019) or with the major investment of energy on defence mechanisms rather than in biomass production (Fahad et al., 2015). In addition, our findings showed that the source of fertilizers has a significant effect but depends also on the dose of P. Poly-B fertilizer significantly increased shoot DW (156.8%) at both doses followed by Ortho-A (125.6%) and Ortho-B (114.2%) at 45 and 30 ppm of P, respectively (Fig 4. A). Furthermore, compared to OrthoP fertilizers, Poly-B showed the best performance at 30 ppm for root DW with an increase of 49.4% and 98.9% compared to C+ and C-, respectively. Therefore, an optimal P-supply stimulated vegetative growth and the creation of strong root systems which is primordial to the efficient absorption of soil nutrients (Sharma et al., 2020). In addition, the effect could be related to the improved P availability in the soil solution due to the slow and continuous release property of polyphosphate. However, a high dose of P-soluble fertilizers (60 ppm) had detrimental effects on salt-stressed wheat (data not shown). In this regard, a harmful reverse effect of high phosphorus dose was also reported in other crops such as common bean (Bargaz et al., 2016), Barley (Zribi et al., 2011) Soybean (Phang et al., 2009) and Maize (Tang et al., 2019). The partitioning of biomass could be regarded as a process for growth optimisation. Balanced growth of both roots and shoots might be a strategy to improve plant productivity in salty soil, which leads to optimal allocation (Hermans et al., 2006) and enhances both P-uptake and water acquisition (Fujita et al., 2004, Meng et al., 2021).In our study, the K content was similar in the roots of fertilized and unfertilized salt-stressed plants (C+) (**Figure 5**), but we noticed a reduction (-20%) in the concentration of potassium in the aerial part in salt-stressed and fertilized plants (C+) (**Figure 6**). The difference between P fertilizers was not significant since we equalized the amount of K for all treatments. Remarkably, it was found that salinity caused sodium injury, which impacts potassium uptake by root cells (Conde et al., 2011; Rahimi et al., 2021). Accordingly, the Na concentration significantly increased in the root and shoot of fertilized plants compared to C+ which was unexpected. The effect was more relevant using Ortho-A at 30 ppm of P with an increase of 28% and 42% in Na accumulation in shoots and roots, respectively (**Figures 5**, **6**). Indeed, it is worth noting that potassium and sodium might exist in competition and induce K+ deficiency in the rhizosphere, and depolarization of the plasma membrane also stimulates the K+ outward rectifying channels to mediate the efflux of K+ and the influx of Na+ (Behdad et al., 2021). Additionally, it was reported that many enzymes (including photosynthetic ones) were severely inhibited by sodium at a concentration above 100 mM (about 10 dS/m) (Shabala and Munns, 2017). Furthermore, the enzymes which need potassium as a cofactor are especially sensitive to the high concentration of sodium (Chaves et al., 2011; García-Ortiz et al., 2012). Our findings were consistent with previous works in the literature (Chen et al., 2007; Rodríguez-Martín et al., 2018). However, it is interesting to mention that the reduction in both phosphorus and potassium concentration under high salinity is accompanied by a significant increase in sodium content in root and shoot (Demiral, 2017; Loudari et al., 2020).

Accordingly, Singh et al. (2016) found that P-fertilization supported the formation of a well-developed root system of lentil plants which optimizes their ability to absorb other minerals from the soil such as N, K+, and Ca2+. Consequently, their amounts increased after the phosphorus application (Singh et al., 2016; Loudari et al., 2020). Besides, it has been reported that phosphorus and potassium are implicated in salt stress mitigation in most crops (Bargaz et al., 2016; Chakraborty et al., 2021). Kaya et al., 2013 reported that phosphorus and potassium, and indole-3-acetic acid (IAA) were efficient in enhancing the maize plant’s fitness when subjected to salt stress. Indeed, Rubio et al. (2005) observed in the leaf and root cells of *Zostera marina L*, a Na-dependent high-affinity phosphate transporter in their plasma membrane. In addition, Zribi et al. (2021) reported that phosphorus availability disturbed Na transportation to shoots which were in line with our results related to Poly-B response to Na accumulation in shoots and roots compared to OrthoP. Accordingly, P fertilizers exhibit similar responses in the total P (Pt) content in the root and shoot of fertilized plants mainly at 45 ppm of P. The increase reached 104% in Root-Pt content in comparison with C+ (**Figure 5**). The same tendency was observed for shoot-Pt content where OrthoP fertilizers showed similar results for both doses (62% and 115% at 30 and 45 ppm of P, respectively) (**Figure 6**). The response was dose-dependent. Moreover, Poly-B fertilizer showed the highest Pt concentration in shoots estimated at 84% and 131% at 30 and 45 ppm of P, respectively (**Figure 6**). Indeed, the rise in P-content in fertilized wheat plants under salinity (**Figures 5**, **6**) could be attributed to a synergistic effect of Na, which is implicated in P acquisition and/or transportation to the aerial part of plants (Grattan and Grieve, 1992). However, high external phosphorus enhanced sodium acquisition and reduced the soybean tolerance to salinity (Phang et al., 2009). This is consistent with our results at 12 WAS, the sodium in shoots of salt-stressed and fertilized plants was significantly higher than in plants grown under salinity and phosphorus deficiency (C+) (**Figures 5**, **6**). Besides, a special partitioning of sodium ions between shoot and root was observed (Keisham et al., 2018). Our findings agree with this statement since we found that Na+ accumulation was important in roots of salt-stressed and fertilized plants compared to shoots (**Figures 5**, **6**). For instance, Na content in plants fertilized by Ortho-A increased by 28% in shoots at 30 ppm of P while it reached + 42% in roots compared to C+ plants. It has also been shown that the decrease in growth under salinity might be attributed to a nutritional imbalance and excessive sodium acquisition (Isayenkov and Maathuis, 2019). Furthermore, it has been reported that photosynthetic and respiratory electron transport were inactivated by sodium accumulation (Stirbet, 2012), which was revealed in our results by a reduction in PI _tot_ (**Figure 3**) and I-P phase (loss of PSI reaction centres) in salt-stressed and unfertilized plants (C+) (**Figures 2A, B**). In the present work, the diminution in J-I-P fluorescence yield (**Figure 2A**) was more significant for salt-stressed and unfertilized plants (C+) in comparison with fertilized ones. These results suggest a restriction of both donor and accepter-side of PSI (reduced J-I-P yield), which indicates that P in wheat leaves stimulated the intersystem electron transport regulation between PSII and PSI (El-Mejjaouy et al., 2022). This could reveal a cellular adaptation to alleviate the harmful effect of salt stress and ensure photosynthetic electron transport equilibrium (Kalaji et al., 2016; Loudari et al., 2020; Muhammad et al., 2021). The P doses and fertilizers forms showed a significant effect on PI_tot_. Compared to C+, the PI_tot_ increased by 128.5%, 90.2% and 38.8% for Ortho-B, Ortho-A and Poly-B, respectively for both doses. Here, the dose did not affect the PI_tot_ but the P supply as OrthoP showed positive responses compared to Poly-B. Indeed, the mild salt tolerance of plants could be partially attributed to their faculty to maintain photosynthetic ability (Sharma et al., 2020) since P is implicated in the transport of fixed carbon from chloroplasts to the cytosol with its triose-phosphate form (Rychter et al., 2018), together with a lower sodium concentration and a higher cytosolic potassium/sodium ratio (Rahimi et al., 2021). Under moderate stress, a small decrease in stomatal conductance could provide protective effects against salinity, through limited water loss and improved plant water-use efficiency (Wilkinson and Davies, 2002). These phenomena restrict CO2 influx and water vapour efflux mainly for C3 plants. Besides, Koyro (2006) reported that the stomatal closure could be considered an adaptive mechanism to mitigate salt stress, rather than its negative consequence. In our study, the P dose of soluble P fertilizers showed a significant effect on stomatal conductance (SC) while the fertilizers forms did not affect this physiological parameter. Indeed, compared to C+, Poly-B and Ortho-A showed similar results in SC with an increase of 157% and 217% at 30 and 45 ppm of P, respectively. The highest significant value of SC was obtained with Ortho-B fertilizer at 45 ppm with an increase of 232% and 56% compared to C+ and C- plants, respectively. The increased SC in salt-stressed plants grown under sufficient P supply leads to an improvement in plant salt tolerance (Behdad et al., 2021). In the present work, Root Tissue Water Content (TWC) (**Figure 4B**) significantly decreased in salt-stressed and unfertilized plants (C+) mainly at 12 WAS compared to fertilized plants under saline conditions. The root TWC increased by 33.7% for Ortho-B and Poly B with a similar response for both doses compared to C+ followed by Ortho-A which showed a response dose-dependent (23.5% and 16.73% at 45 and 30 ppm of P, respectively). These findings support the previous work of Li et al. (2009), who revealed that P shortage altered root hydraulic conductance and lowered plant water potential by reducing the water channel proteins activity: the aquaporins. Additionally, our results showed that the shoot water content in salt-stressed and unfertilized plants (C+) has not been reduced under salinity, but this parameter has been significantly decreased for other P-treatments. This response was not strongly influenced by forms or doses of P-fertilizers (**Figure 4B**). Our results are in accord with those of Zribi et al. (2021) on *Aeluropus littoralis* plants. In addition, Asrar et al. (2017) reported that stomata closure can lead to low photosynthetic rates, but our data showed that although the salt-stressed and unfertilized wheat plants (C+) presented a higher TWC of shoots compared to salt-stressed and fertilized plants (+36%) (**Figure 4B**), the performance index (PI_total_) increases significantly for all rates and forms of P in comparison with C+ plants at 6, 8, 10 and 12 WAS (**Figure 3**). Thus, the disturbed water potential under salt stress could not be the cause of the reduced photosynthetic performance. Instead, many studies proposed that diffusional constraints are the main reason for photosynthesis inhibition (Pérez-López et al., 2012; Chen et al., 2015). The downregulation of the photosynthetic metabolism leads to leaf biochemistry variations in response to a reduction in net CO_2_ assimilation under prolonged stresses (Chaves et al., 2011). Prolonged exposure to salt stress or/and P deficiency disturbs biochemical processes (e.g., the activity of Rubisco and Ribulose-1,5-bisphosphate (RuBP) and triose phosphates regeneration) which control gas exchange (Meng et al., 2021). Additionally, several studies showed that P deficiency disturbs ultimately CO2 assimilation (Rychter et al., 2018; El-Mejjaouy et al., 2022). Besides, it has been reported that in response to the decrease in CO_2_ concentration in leaf intercellular airspaces, the activity of other enzymes (Sucrose phosphate synthase (SPS) or nitrate reductase) was reduced (Muhammad et al., 2021). Indeed, under those conditions limiting the fixation of CO_2_, the rate of reduction of energy production is greater than the rate of its use by the Calvin cycle. This might create competition for the use of the energy absorbed during stress, resulting in a reduction in the quantum yield of PSII (Wieneke et al., 2022). Previous studies have shown that adequate phosphate nutrition is crucial for the efficient compartmentation of ions by contributing to the effective partitioning of carbon and the use of photo-assimilates in salt-stressed wheat (Khan et al., 2013; Abbas et al., 2018). Chlorophyll content reduction was observed in salt-stressed and unfertilized plants (C+) (**Figure 1A**). This reduction could be caused either by the limitation in the biosynthesis of chlorophyll or the degradation of existing chlorophyll (Ashraf and Harris, 2013; Carstensen et al., 2018), induces structural variations in the light-harvesting complex, disturbs light fixation ability and reduces photosynthetic efficiency (Duarte et al., 2013; Meng et al., 2021), while a higher chlorophyll content in fertilized plants promotes photosynthetic activity, intensive growth and higher biomass yield (Mohamed et al., 2021). This statement confirms our findings in plants treated with soluble fertilizers under saline conditions where the CCI increased by 93%, 81% and 71% in plants fertilized by Poly-B, Ortho-B and Ortho-A, respectively, compared to C+ (**Figure 1A**). The P doses did not show a significant effect on CCI while the difference between fertilizer forms was significant mainly for Poly-B which increased by 17.42% at 30 ppm f P compared to Ortho-A and Ortho-B at 45 ppm of P.

## 5 Conclusion

This study focused on different growth and physiological responses of wheat grown under the combined effect of salt stress and phosphorus availability using different rates and forms of soluble P-fertilizers. Furthermore, our work shows the relative contribution of stomatal, photochemical, and biochemical factors in restricting plant growth and the photosynthetic performance of durum wheat under salt stress. The results obtained have demonstrated that phosphorus fertilization significantly improved photochemical activity, which was due to enhanced light energy absorbed by enhanced Chl antenna to improve CO_2_ assimilation rate and increased all other growth parameters of the salt-stressed wheat plants. Compared to OrthoP Poly-B fertilizer showed the best performance. Poly-B enriched soil with high quantities of available P which positively impacts the P uptake by plants grown under salinity. The slow and continuous release of available P in the soil and the property of chelating micronutrients make PolyP a promising alternative to reduce the frequency of P application for effective management of P fertilization under salt stress with a higher yield.

## Data availability statement

The original contributions presented in the study are included in the article/supplementary materials. Further inquiries can be directed to the corresponding author.

## Author contributions

AL and AO conceptualized and designed the experiment and lab studies. AL and AM performed the studies. AL, AO, and GC analysed the samples and data. AL, YZ, and AO, wrote the paper. All authors contributed to the article and approved the submitted version.

## Acknowledgments

OCP Group and Prayon, the SoilPhorLife project sponsors, are greatly acknowledged for funding this study. The authors also thank Mrs. Sabah Fathallah and Mr. Aziz Soulaimani for their help and support in sample chemical analysis. The UM6P language laboratory team is acknowledged for proofreading the article.

## Conflict of interest

The authors declare that the research was conducted in the absence of any commercial or financial relationships that could be construed as a potential conflict of interest.

## Publisher’s note

All claims expressed in this article are solely those of the authors and do not necessarily represent those of their affiliated organizations, or those of the publisher, the editors and the reviewers. Any product that may be evaluated in this article, or claim that may be made by its manufacturer, is not guaranteed or endorsed by the publisher.

## Abbreviations

CCI, Chlorophyll content index; CEC, Cation exchange capacity; Chl, Chlorophyll; Corg, Organic carbon; DTPA, Diethylene triamine penta acetic acid; DW, Dry weight; e-, electron; EC, Electrical conductivity; FW, Fresh weight; IAA, Indole-3-acetic acid; NT, Total Nitrogen; OEC, Oxygen-evolving complex; OM, Organic matter; PGPR, Plant-growth-promoting rhizobacteria; PIABS, Photosynthetic performance index; PItot, Photosynthetic performance; PSB, Phosphate solubilizing bacteria; PSI, Photosystem I; PSII, Photosystem II; Pt, Total Phosphorus; RC, Reaction centres; RDW, Root dry weight; ROS, Reactive oxygen species; RuBP, Ribulose-1,5-bisphosphate; SC, Stomatal conductance; SDW, Shoot dry weight; SPS, Sucrose phosphate synthase; TWC, Tissue water content; WAS, Week after sowing; WUE, water use efficiency; φPo, Quantum yield of electron transport.
